# Overweight, Obesity, and Perception of Body Image Among Slum Residents in Nairobi, Kenya, 2008–2009

**DOI:** 10.5888/pcd10.130198

**Published:** 2013-12-19

**Authors:** Remare Ettarh, Steven Van de Vijver, Sam Oti, Catherine Kyobutungi

**Affiliations:** Author Affiliations: Steven Van de Vijver, Sam Oti, Catherine Kyobutungi, African Population and Health Research Center, Nairobi, Kenya.

## Abstract

**Introduction:**

The increase in cardiovascular diseases in sub-Saharan Africa has been attributed in part to the changes in lifestyle, and the prevalence of risk factors for cardiovascular disease is higher among urban populations than among nonurban populations. The objective of this study was to determine the prevalence of overweight and obesity and examine perceptions of body size differentiated by sex and other determinants among slum dwellers in Nairobi, Kenya.

**Methods:**

Analysis included 4,934 adults randomly selected from the Korogocho and Viwandani slums of Nairobi. Height and weight were measured during interviews; body mass index (BMI) was calculated. Perceptions of current and ideal body image were determined by using 18 silhouette drawings of body sizes ranging from very thin to very obese. We used multivariate logistic regression analysis to determine predictors of underestimation of body weight among overweight and obese respondents.

**Results:**

Overall, 43.4% of women and 17.3% of men in the study population were overweight or obese. More than half (53%) of those who were overweight or obese underestimated their weight; 34.6% of women and 16.9% of men did so. In all BMI categories, more than one-third of women and men preferred body sizes classified as overweight or obese.

**Conclusion:**

This study highlights the prevalence of overweight and obesity and the strong preference for larger body size among adults in the slums of Nairobi. Interventions to educate residents on the health risks associated with excess body weight are necessary as a part of strategies to reduce the prevalence of risk factors for cardiovascular disease in these settlements.

## Introduction

Cardiovascular disease has become the leading cause of death in sub-Saharan Africa (1). Increasing urbanization and associated lifestyle changes may induce risk factors, such as obesity, for cardiovascular disease (2,3). In sub-Saharan Africa, the rise in the prevalence of obesity in urban areas has coincided with the growth of an increasingly educated and wealthier middle class that engages in less physical activity and consumes greater amounts of calorie-dense foods than the poorer segments of the population (3,4). However, the change in lifestyle in urban sub-Saharan Africa also profoundly affects the urban poor, and the prevalence of risk factors for cardiovascular disease is high in resource-deprived city slums (4,5). Although studies show that obesity is increasing among the urban poor (4), data are limited on the extent of the problem among city slum populations in different countries in sub-Saharan Africa.

Cultural ideals influence how people assess their body image and body weight (6,7). Studies in sub-Saharan Africa suggest that the poor tend to perceive body size and its health implications differently from people in the same cities with greater wealth and education (8). In some societies in sub-Saharan Africa, a larger body size is commonly assumed to reflect good health and higher social status and may thus be considered desirable (8,9). In addition to examining culturally desired body sizes among the poor in sub-Saharan Africa, it is also relevant to learn how people perceive their own body size and weight, because underestimation of body mass index (BMI) is prevalent in low-income settings (10) and may predict overweight or obesity (11). Although some of the factors that influence perception of body image have been studied in developed countries, this topic remains largely unexplored in sub-Saharan Africa. Body image perceptions and preferences differ by sex in the United States (12,13), but the effects of age and marital status are unclear (14,15). The objective of this study was to determine the prevalence of overweight and obesity and examine perceptions of body size differentiated by sex and other determinants among slum dwellers in Nairobi, Kenya.

## Methods

### Study participants and design

The study was part of a cross-sectional survey designed to assess the linkages between socioeconomic and sociocultural factors, perceived personal risk for cardiovascular disease, and health behavior in the Korogocho and Viwandani slums of Nairobi. The survey was conducted from May 2008 through April 2009 as part of the Nairobi Urban and Health Demographic Surveillance System (NUHDSS), which is run by the African Population and Health Research Center. The 2 slums are typical of the informal settlements in Nairobi, although they differ in terms of community and population stability. Korogocho is populated by more settled residents, many of whom have lived there for many years. Viwandani, on the other hand, has a more a youthful and transient population that is drawn to the location by job opportunities in the nearby industrial area. However, the levels of poverty are high in both slums, and incomes are lower than those in non-slum areas of the city (16). Characteristics of the 2 slums and details of the NUHDSS and its operations are available elsewhere (16).

A total of 5,190 residents of the 2 slums were randomly selected and stratified by sex and age using a sampling frame that included all adults aged 18 years or older in the NUHDSS. Overall response rates were 94% in Korogocho and 95% in Viwandani. All interviews and measurements were conducted by trained field workers using a structured questionnaire translated into Kiswahili. The purpose of the study was explained to each respondent and signed consent obtained before the interview was conducted and anthropometric measurements taken. The study protocol was approved by the Kenya Medical Research Institute/National Ethical Review Committee (NON-SSC Protocol No.339).

### Study variables

The participants were administered a questionnaire that included sections on sociodemographic characteristics. Age of respondents was obtained as a continuous variable in years and categorized into age groups. Sex, marital status, and educational attainment were reported by each respondent, while socioeconomic status was determined as an index based on ownership of household assets and categorized into quintiles (16). The weight of each respondent was measured to the nearest 0.1 kg and height to the nearest centimeter using a SECA electronic digital weighing scale and a SECA portable stadiometer (Seca GmbH, Hamburg, Germany). The reliability of all measurements between field workers was assessed during training and piloting of the questionnaire. BMI was calculated as body weight in kilograms divided by the square of the height in meters (17). BMI was categorized according to World Health Organization classifications (17): underweight (BMI <18.5), normal (BMI = 18.6–24.9), overweight (BMI = 25.0–29.9), and obese (BMI ≥30.0). 

**Figure 1:**
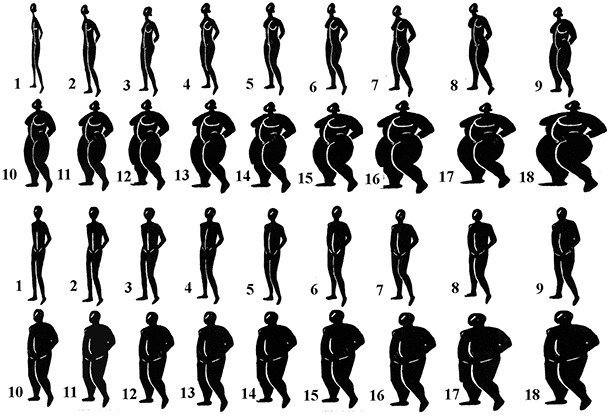
Body image rating scale for men and women. Images 1 through 5 represent people who are underweight, images 6 through 9 represent people of normal weight, images 10 through 13 represent overweight people, and images 14 through18 represent obese people (11).

Body image was assessed by using methods developed for studies of body image and eating disorders (18). The body image rating scale included 18 silhouettes of each sex, ranging from very thin to very obese (Figure 1). This validated scale is widely used, and body images correlate strongly with objective measures of body size (19,20). Each figure was displayed on cards 16 cm in height, and the randomly numbered cards were displayed to participants in a random order. To determine the current body image, participants were asked, “Which image most accurately depicts your current body size?” To determine ideal body size, the participant was asked, “Which image most accurately depicts the body size you would wish to have?” The 18 images were divided into 4 categories by adapting the image scheme described by Madrigal et al (11). 

### Data analysis

Descriptive analysis of BMI, current body image, and ideal body image by sex was conducted by using proportions. The proportion of respondents underestimating their weight in each BMI category was calculated for both sexes. The extent of overestimation of, agreement with, or underestimation of actual body size was determined by comparing the BMI category with the current body image category. To determine predictors of underestimation of BMI among overweight or obese participants, a multivariate logistic regression model was fit using sociodemographic variables that were significant (at *P* < .25) in the univariate logistic analysis. The dependent variable was a binary outcome of whether or not the participant underestimated his or her BMI category (underestimation = 1; agreement or overestimation = 0). The independent variables in the model were age, sex, marital status, and education. The bivariate relationship between BMI and ideal body image was determined. All statistical analyses were done by using Stata version 11 (Stata Corp, College Station, Texas).

## Results

Of the 5,190 people recruited into the main study, 4,934 people (comprising 2,669 [54%] men and 2,265 [46%] women) had the data for the required variables and were included in the analysis. The mean age of the respondents was 42 years: 45% of respondents were younger than 40 years, and 55% were aged 40 years or older. The proportion of the respondents who had primary education or less was 75%, and approximately 2.5% had postsecondary education. 

Overall, 43.4% of women and 17.3% of men in the study population were overweight or obese (Table 1). Obesity was more prevalent among women (15.5%) than among men (2.3%). Approximately half (50.5%) of women rated their current body image as normal weight; although 14.2% rated their current body image as underweight, only 5.1% were underweight according to BMI. Among men, 52.7% rated their current body image as normal weight; according to BMI, 72.9% were normal weight. The proportions of men who perceived themselves to be overweight (20.8%) or obese (13.4%) were greater than the proportions who were overweight or obese according to BMI (15.0% and 2.3%, respectively). Among women, the distribution of categories for ideal body image was not substantially different from the distribution of categories for BMI. For example, 14.8% of women chose an obese image as ideal, and 15.5% of women were obese. These distributions, however, were different among men: 20.6% of men chose an obese image as ideal, whereas only 2.3% of men were obese according to BMI.

**Table 1:** Distribution by Weight Category According to Body Mass Index, Current Body Image, and Ideal Body Image in Women and Men, Nairobi, Kenya, 2008–2009^a^

Category	Women (n = 2,265)			Men (n = 2,669)		
	Body Mass Index	Current Body Image	Ideal Body Image	Body Mass Index	Current Body Image	Ideal Body Image
Underweight	115 (5.1)	321 (14.2)	160 (7.1)	262 (9.8)	352 (13.2)	162 (6.1)
Normal weight	1,167 (51.5)	1,143 (50.5)	1,209 (53.4)	1,946 (72.9)	1,406 (52.7)	1,104 (41.3)
Overweight	631 (27.9)	503 (22.2)	562 (24.8)	401 (15.0)	554 (20.8)	854 (32.0)
Obese	352 (15.5)	298 (13.2)	334 (14.8)	60 (2.3)	357 (13.4)	549 (20.6)

a All values are no. (%). All percentages are based on column totals. Percentages may not add up to 100 because of rounding.

**Figure 2:**
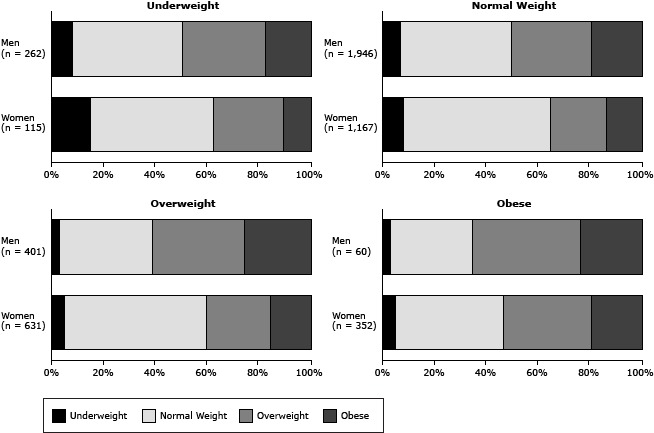
Ideal body image, by current body mass index (BMI) and sex. Survey participants were asked to indicate the body size they wished to have. **Table:** 

In all BMI categories, more than one-third of women preferred body images classified as overweight or obese, and the proportion of men with a preference for overweight or obese body images was greater than that of women (Figure 2). More than half of women and men classified as overweight or obese indicated a preference for overweight or obesity.

Women		Men	
Ideal body image	No. (%)	Ideal body image	No. (%)
**Underweight (n = 115)**		**Underweight (n = 262)**	
Underweight	17 (15)	Underweight	20 (8)
Normal weight	55 (48)	Normal weight	112 (43)
Overweight	31 (27)	Overweight	84 (32)
Obese	12 (10)	Obese	46 (18)
**Normal Weight (n = 1,167)**		**Normal Weight (1,946)**	
Underweight	93 (8)	Underweight	129 (7)
Normal weight	660 (57)	Normal weight	828 (43)
Overweight	256 (22)	Overweight	602 (31)
Obese	158 (14)	Obese	387 (20)
**Overweight (n = 631)**		**Overweight (n = 401)**	
Underweight	34 (5)	Underweight	11 (3)
Normal weight	347 (55)	Normal weight	145 (36)
Overweight	157 (25)	Overweight	143 (36)
Obese	93 (15)	Obese	102 (25)
**Obese (n = 352)**		**Obese (n = 60)**	
Underweight	16 (5)	Underweight	2 (3)
Normal weight	147 (42)	Normal weight	19 (32)
Overweight	118 (34)	Overweight	25 (42)
Obese	71 (20)	Obese	14 (23)

Among women, 18.2% overestimated their BMI; 33.0% of men overestimated their BMI (Table 2).Underestimation was more prevalent among women (34.6%) than among men (16.9%). Obese women accounted for 28.8% of total underestimation among women, whereas obese men accounted for only 7.8% of total underestimation among men.

**Table 2:** Proportion of Women and Men and Their Overestimation of, Agreement With, or Underestimation of Body Mass Index, by Weight Category, Nairobi, Kenya, 2008–2009^a^

Category	Overestimation		Agreement		Underestimation	
	Women	Men	Women	Men	Women	Men
Underweight	60 (14.6)	195 (22.1)	55 (5.1)	67 (5.0)	—	—
Normal	257 (62.4)	588 (66.7)	705 (65.9)	1,105 (82.6)	205 (26.1)	253 (56.2)
Overweight	95 (23.1)	98 (11.1)	183 (17.1)	141 (10.5)	353 (45.0)	162 (36.0)
Obese	—	—	126 (11.8)	25 (1.9)	226 (28.8)	35 (7.8)
**Total^b^**	**412 (18.2)**	**881 (33.0)**	**1,069 (47.2)**	**1,338 (50.1)**	**784 (34.6)**	**450 (16.9)**

a All values are no. (%). Percentages in the table may not add up to 100 because of rounding.

b Percentages for only this row are based on total counts for each sex.

Of 1,444 participants classified as overweight or obese, 776 underestimated their BMI. Of the key sociodemographic characteristics of the participants, sex was a significant predictor of underestimation of BMI among normal-weight participants, whereas age and sex were significant predictors among overweight or obese participants (Table 3). Overweight or obese participants aged 50 years or older were less likely than their younger counterparts to underestimate their BMI. Similarly, men were less likely than women to underestimate their BMI in both age categories. Marital status and education were not significantly associated with the likelihood of BMI underestimation.

**Table 3:** Sociodemographic Predictors of Body Mass Index Underestimation Among Normal-Weight and Overweight or Obese Survey Respondents, Nairobi, Kenya, 2008–2009

Characteristic	Normal Weight			Overweight or Obese		
	No. of Respondents	% Who Under-estimated BMI	OR (95% CI)	No. of Respondents	% Who Under-estimated BMI	OR (95% CI)
**Age, y**						
18–29	819	15.5	1 [Reference]	202	64.9	1 [Reference]
30–39	705	15.9	1.09 (0.80–1.49)	318	60.4	0.90 (0.61–1.33)
40–49	676	13.0	0.87 (0.62–1.23)	398	56.8	0.80 (0.55–1.18)
50–59	551	13.8	0.84 (0.57–1.24)	309	47.9	0.53^a^ (0.35–0.80)
≥60	362	15.2	1.10 (0.68–1.77)	217	36.4	0.38^a^ (0.23–0.63)
**Sex**						
Female	1,167	17.6	1 [Reference]	983	58.9	1 [Reference]
Male	1,946	13.0	0.71^a^ (0.56–0.89)	461	42.7	0.50^a^ (0.38–0.65)
**Marital status**						
Married/cohabiting	2,203	13.5	1 [Reference]	984	53.5	1 [Reference]
Never married	488	17.8	1.24 (0.90–1.71)	142	62.7	1.17 (0.78–1.78)
Ever married	422	17.2	1.21 (0.85–1.72)	318	50.6	0.85 (0.61–1.18)
**Education**						
Less than primary	681	17.3	1 [Reference]	393	55.7	1 [Reference]
Primary	1,373	14.3	0.77 (0.59–1.01)	554	56.7	0.89 (0.67–1.17)
Secondary	619	12.9	0.74 (0.52–1.03)	274	54.0	0.78 (0.55–1.10)
College/university	72	15.3	0.88 (0.44–1.76)	29	58.6	0.97 (0.43–2.17)
**SES quintiles^b^**						
Lowest (poorest)	678	14.9	—	248	50.0	—
Second	484	15.9	—	188	51.6	—
Middle	551	14.7	—	174	56.9	—
Fourth	673	16.3	—	333	52.5	—
Highest (least poor)	635	12.0	—	464	56.6	—

Abbreviations: BMI, body mass index; OR, odds ratio; CI, confidence interval; SES, socioeconomic status.

a *P* < .001 from multivariate logistic regression.

b Socioeconomic status was excluded from the model because of lack of significance in the univariate logistic regression analysis.

## Discussion

Our study was designed to investigate the prevalence of overweight and obesity, the extent of disagreement between perceived body image and BMI, and the patterns of preferred body size in 2 slums in Nairobi. The prevalence of overweight or obesity among women in these urban slums was similar to the prevalence reported in a national survey of urban women in Kenya (4), and the sex pattern shown in our study confirms the patterns reported in other studies in Africa (21,22). In the context of poverty and resource deprivation in slums, overweight and obesity are a challenge and help to drive the increasing rates of morbidity associated with cardiovascular disease in these urban settlements (23–25).

A major issue associated with overweight and obesity is the perception of body size in different cultural settings (26,27). Our study used body images or silhouettes to estimate self-reported body size. Differences between objective measures of body size (ie, BMI) and individual perception of current body image suggest individual or societal definitions of desirable or “normal” body size that are not based on medical facts. Our study found that more than half of the population who were overweight or obese underestimated their weight, suggesting that most of these people do not acknowledge their extra weight and the related health risks.

We also determined the ideal body image for each survey participant and how it related to their actual BMI category. Regardless of BMI category, substantial proportions of women and men indicated a desire to have a body size that was larger than their actual size. More than half of the participants who were overweight or obese chose an ideal body image that was either overweight or obese. Families, communities, and society strongly influence perceptions of body image, and excess body weight has generally been associated with wealth and health and considered a desirable attribute in many parts of sub-Saharan Africa (6,8,22). The implications of excess body weight as a risk factor for noncommunicable diseases are not readily apparent to most residents of poor settlements because of low levels of education and the lack of emphasis on noncommunicable diseases in traditional health education and counseling at the community level in sub-Saharan Africa.

In our focus on overweight and obese participants, we sought to identify sociodemographic characteristics associated with underestimation of BMI. More than half (54%) of this subgroup underestimated their BMI, and age and sex were the only significant predictors of underestimation. These results are consistent with reports of greater dissatisfaction with body image among younger adults and women (28). Although studies have found that education and income are associated with the likelihood of obesity in urban sub-Saharan Africa (4), we could not find reports on the determinants of underestimation of BMI in this high-risk subgroup.

This study has several limitations. One is the use of a discrete number of silhouettes to assess body image. By using this method, body size is considered a continuous variable measured from a finite number of images (29). The amount of information lost by using this method can be reduced by increasing the precision of the instrument. In our study, we attempted to reduce the coarseness of the scale by using an 18-silhouette scale developed and validated in a previous study (30). Other limitations of the study include the challenge of establishing the influence of cultural preferences and beliefs on body image in slum settlements comprising diverse ethnic groups and the extent to which these 2 slums are representative of other slums in Nairobi. A strength of the study is the participation of a large number of residents of an informal urban settlement in Kenya. Although a large proportion of the urban population in sub-Saharan Africa lives in slums, data are limited on the increasingly relevant topic of obesity and weight-related perceptions among this growing group.

Our study has important implications for the effort to reduce the prevalence of risk factors for cardiovascular disease in poor urban settings in Kenya and the rest of sub-Saharan Africa. As the epidemiological transition continues among low- and middle-income countries, the prevalence of obesity also increases, even among low-income groups. The high levels of overweight and obesity reported in our study — despite the high levels of poverty found in the slums we studied — suggest that effective dietary and lifestyle interventions are needed to address this issue in slums. Innovative strategies to ensure better appreciation and understanding of the health consequences of overweight and obesity by slum residents are required if efforts to reverse the greater preference for larger body size are to be successful.
